# Thyrotoxic Cardiomyopathy Unveiled: Insights From a Compelling Case Report

**DOI:** 10.7759/cureus.51172

**Published:** 2023-12-27

**Authors:** Anas Ibraheem, Abdullah Abdullah

**Affiliations:** 1 Internal Medicine/Clinical Hematology, Al Karama Teaching Hospital, Baghdad, IRQ; 2 General Medicine, Frimley Health NHS Foundation Trust/Wexham Park Hospital, Slough, GBR

**Keywords:** heart failure with preserved ejection fraction (hfpef), cmri cardiac magnetic resonance imaging, hyperthyroidism, heart failure, thyrotoxic cardiomyopathy, thyrotoxic cardiovascular disease, thyrotoxicosis

## Abstract

Thyrotoxicosis is a clinical condition characterized by inappropriately elevated thyroid hormone levels in the bloodstream, leading to systemic effects on the body. In fact, the thyrotoxic state has tight regulatory control over the cardiovascular system through genomic and non-genomic mechanisms. This study highlights a rare presentation of thyrotoxic cardiomyopathy (TCM), which, to the best of our knowledge, is one of the very few case reports involving heart failure with preserved ejection fraction (HFpEF) and only atrial involvement, compared to the previous literature.

A 37-year-old female presented to the outpatient clinic with abdominal distention and neglected signs and symptoms consistent with thyrotoxicosis for a year. Investigations revealed high N-terminal pro-b-type natriuretic peptide (NT-proBNP) levels of 1788 pg/mL. Cardiac MRI and trans-thoracic echocardiogram (TTE) revealed bilateral atrial dilatation, a left ventricular ejection fraction (LVEF) of 60%, and diastolic dysfunction. Additionally, severe free-flowing tricuspid and mitral valve regurgitation were observed, with no evidence of pericardial effusion or ventricular abnormalities. Therefore, a diagnosis of TCM was suspected and eventually confirmed by excluding other differential diagnoses. Besides a diffuse goiter on ultrasonography, the thyroid panel test revealed low thyroid-stimulating hormone (TSH) levels of <0.01 mIU/L, a free thyroxine T4 of >100 pmol/L, and positive anti-thyroid peroxidase (TPO) and TSH receptor antibodies. Accordingly, a team of endocrinologists, cardiologists, and internists managed the patient with anti-thyroid medications alongside symptomatic treatment. A few days later, she was discharged in good condition, and a follow-up visit was arranged with the endocrinology and cardiology clinics.

It is crucial to maintain a high level of suspicion to detect and treat TCM promptly, and a multidisciplinary approach should ideally be employed. This is not only important for the prevention of but also reversing potentially life-threatening cardiovascular complications.

## Introduction

Thyrotoxicosis is a clinical condition marked by inappropriately elevated thyroid hormone levels of tri-iodothyronine (T3) and/or thyroxine (T4) in the bloodstream, leading to various systemic effects on the body [1]. The thyroid gland and cardiovascular system are no exceptions, having a well-established connection. Studies have shown that the thyrotoxic state has a tight regulatory control over the cardiovascular system. Therefore, the umbrella term of "cardio-thyrotoxic syndrome" has been used to refer to various cardiovascular complications resulting from thyrotoxicosis, as well as hyperthyroidism. These complications can include an increased risk of atrial fibrillation (AF), cardiovascular disease (CVD), heart failure (HF), and, although rare, thyrotoxic cardiomyopathy (TCM) [2-4].

TCM is a diagnosis of exclusion, and the goal of treatment is to achieve euthyroidism to have a better prognosis and cardiovascular outcomes in terms of heart rate and cardiac output [4]. Thus, it is imperative to maintain a high level of suspicion for thyroid disorders in patients with new-onset atrial fibrillation, heart failure, and tachycardia-induced cardiomyopathy. The most effective management strategy to control these patients' blood pressure and heart rate involves using anti-thyroid medications and treating any possible cardiovascular complications [5,6].

Among the numerous articles that have explored TCM in the literature [5,7-11], this study, to the best of our knowledge, is one of the very few case reports of TCM presenting as heart failure with preserved ejection fraction (HFpEF) and only atrial involvement. Our findings provide a unique perspective that will advance the understanding of this rare condition and could lead to improved diagnosis and treatment for these patients.

## Case presentation

A 37-year-old female presented to the outpatient clinic with abdominal distention after a mechanical fall while cleaning the stairs five days earlier. Apart from a minor cut in her right eyebrow, there were no associated injuries. On further questioning, her sister stated that she did not have time to do regular check-ups or visit her general practitioner due to her busy schedule as a house cleaner. Therefore, she had been neglecting symptoms of increasing abdominal girth, palpitations, weight loss, and frequent bowel motions for a year and linking them to her work stress. She had a past medical history of depression and mechanical back pain, which were treated accordingly with citalopram and co-codamol. Regarding her social history, she was a light daily smoker and social drinker, lived with her family, and was usually independent.

On physical examination, she was found to be diaphoretic and underweight, with signs of exophthalmos and fine bilateral hand tremor. Her vital signs were unremarkable, except for a resting pulse rate of 148 beats per minute with an irregular rhythm. The abdominal examination revealed apparent abdominal distention with striae, shifting dullness, and transmitted thrill. Hepatomegaly was present, with mild tenderness in the right upper quadrant. The neck examination showed an anterior painless mass, raised jugular venous pressure (JVP), and visible carotid pulsations. Her chest examination was unremarkable, except for a non-radiating pansystolic murmur best heard over the tricuspid region.

**Figure 1:**
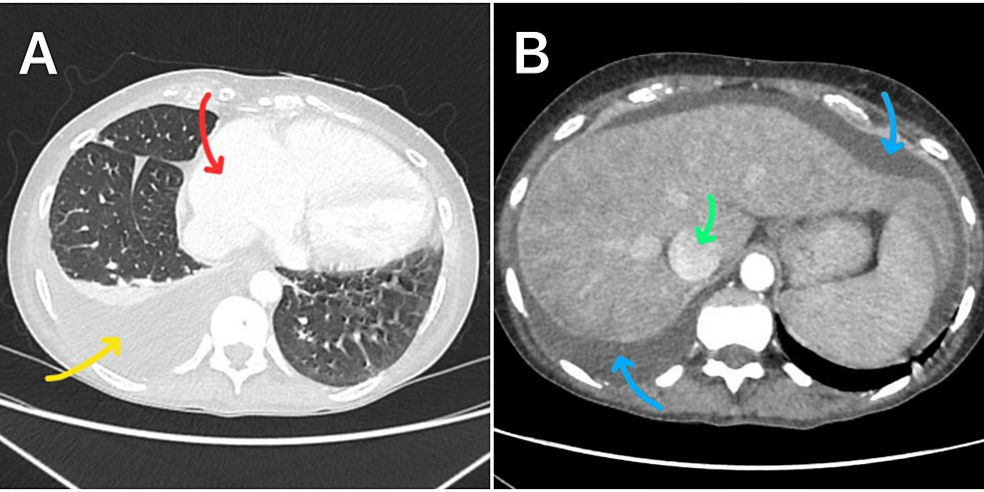
Initial CT scan of the chest (A) and abdomen (B) The images show a moderate right-sided pleural effusion (yellow arrow), cardiomegaly with bilateral atrial dilatation (red arrow), moderate ascites (blue arrows), and hepatomegaly with prominent dilatation of the inferior vena cava (green arrow) without any evidence of liver diseases such as cirrhosis, hepatocellular carcinoma, or Budd-Chiari syndrome, making the diagnosis of passive hepatic congestion more likely CT: computed tomography

Given the possibility of internal organ injury causing fluid in the peritoneum, she underwent abdominal ultrasonography and a trauma CT scan, which did not show any organ injury; however, moderate ascites, hepatomegaly, enlarged inferior vena cava, right-sided pleural effusion, and cardiomegaly were observed (Figure 1).

**Figure 2:**
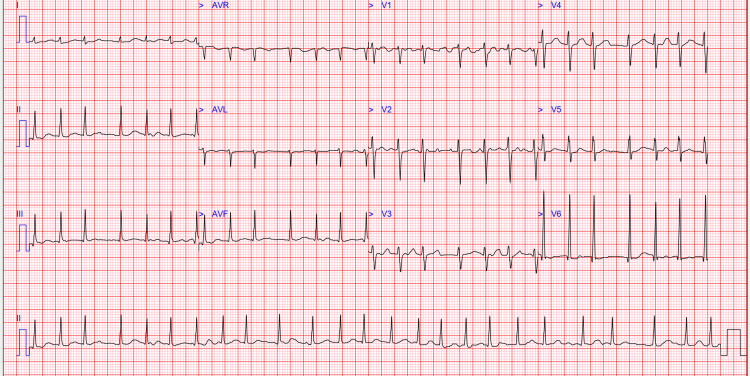
Initial electrocardiogram showing atrial fibrillation

In addition, her initial blood investigations were normal for complete blood count, kidney function, virology screen, and transaminases, but alkaline phosphatase (ALP) was elevated. The coagulation screen showed high D-dimer levels, mild elevation of prothrombin time (PT), and normal activated thromboplastin time (APTT) (Table 1). Fast AF was confirmed on ECG (Figure 2).

**Table 1:** Summary of the patient’s laboratory data on admission MCV: mean corpuscular volume; MCH: mean corpuscular hemoglobin; MCHC: mean corpuscular hemoglobin concentration; PT: prothrombin time; INR: international normalized ratio; PTT: partial thromboplastin time; ALP: alkaline phosphatase; ALT: alanine aminotransferase; AST: aspartate aminotransferase; T3: triiodothyronine; T4: thyroxine; TSH: thyroid-stimulating hormone; BUN: blood urea nitrogen; Na: sodium; K: potassium; CRP: C-reactive protein; NT-proBNP: N-terminal pro b-type natriuretic peptide; TSH receptor antibodies: thyroid-stimulating hormone receptor antibodies; TPO antibodies: thyroid peroxidase antibodies

Test	Result	Reference range	Test	Result	Reference range
White blood count	6.1 × 10^9^/L	4.5–11 × 10^9^/L	Albumin	37 g/dL	34–54 g/L
Hemoglobin level	12 g/dL	11.5–16 g/dL	ALP	287 U/L	35–104 U/L
MCV	81 fL	80–100 fL	ALT	19 U/L	10–35 U/L
MCH	29.5 pg	27–32 pg	AST	23 U/L	10–35 U/L
MCHC	34 g/dL	32–36 g/dL	BUN	4.2 mg/dL	5–20 mg/dL
Platelet count	140 × 10^9^/L	145–400 × 10^9^/L	Creatinine	0.9 mg/dL	0.5–1.1 mg/dL
D-dimer	3.4 μg/mL	≤0.49 μg/mL	Na	139 mmol/L	135–145 mmol/L
PT	18.5 seconds	11.8–14.4 seconds	K	3.8 mmol/L	3.5–5 mmol/L
INR	1	0.87–1.13	Calcium	2.2 mmol/L	2.15–2.55 mmol/L
PTT	37.1 seconds	24.4–36.6 seconds	Phosphate	1.46 mmol/L	0.81–1.45 mmol/L
T3	6.5 pmol/L	2–7 pmol/L	Total bilirubin	21 mg/dL	1.71–20.5 µmol/L
T4	>100 pmol/L	12–30 pmol/L	TSH receptor antibodies	18 IU/L	0–0.9 IU/L
TSH	<0.01 mIU/L	0.3–4 mIU/L	TPO antibodies	432 IU/L	0–34 IU/L
NT-proBNP	1788 pg/mL	<125 pg/mL	CRP	1 mg/dL	0.3–1.0 mg/dL

**Figure 3:**
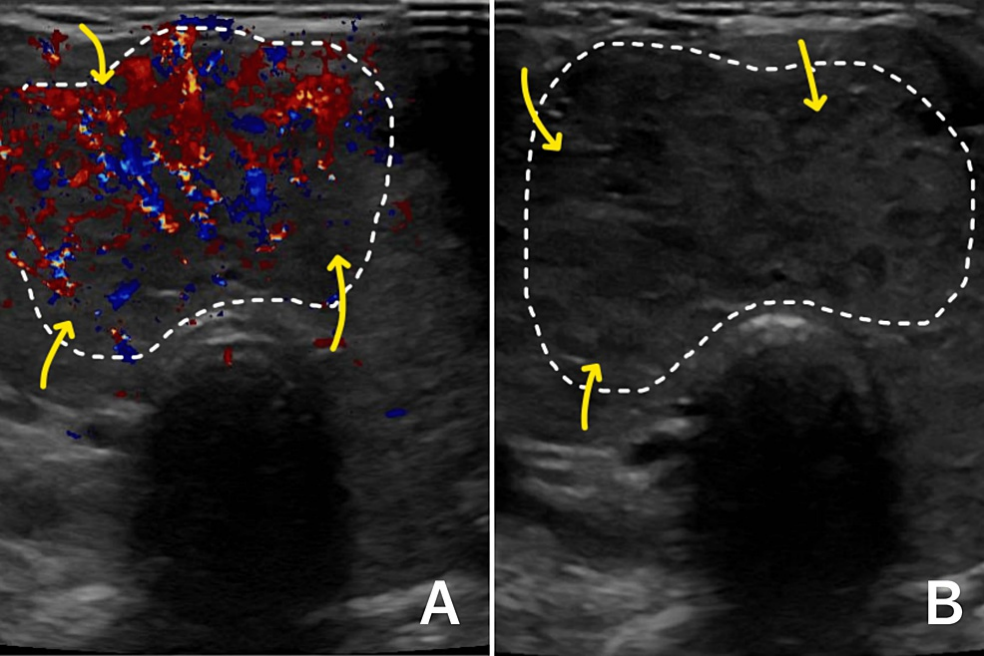
Color Doppler ultrasound of the neck (A) and transverse gray‑scale ultrasound (B) (A) The image demonstrates both central and peripheral hypervascularity of the thyroid gland (yellow arrows). (B) The image shows a diffusely enlarged, heterogeneous, and hypoechoic thyroid mass (yellow arrows), making the diagnosis of Graves' disease more likely

The patient was sent for a thyroid function test, which revealed low thyroid-stimulating hormone (TSH) levels (<0.01 mIU/L) and high free thyroxine T4 (>100 pmol/L). Afterward, neck ultrasonography and further antibody testing for the thyroid gland were done. The results showed a diffusely enlarged, hyperemic, heterogeneous, and hypoechoic thyroid mass resembling thyroiditis with no evidence of thyroid nodules (Figure 3). Positive TSH receptor antibodies and positive anti-thyroid peroxidase (TPO) were found on blood tests. As a result, Graves’ disease was confirmed.

A diagnosis of HF induced by thyrotoxicosis was suspected; therefore, N-terminal pro-b-type natriuretic peptide (NT-proBNP) was tested and showed high levels (1788 pg/mL).

Consequently, a trans-thoracic echocardiogram (TTE) revealed findings consistent with bilateral atrial dilatation with calculated left ventricular ejection fraction (LVEF) of 60% by Simpson's biplane method. Furthermore, severe free-flowing tricuspid and mitral valve regurgitation were observed, with no evidence of pericardial effusion or ventricular abnormalities. On the other hand, the assessment of diastolic dysfunction and left ventricular filling pressure was challenging due to the presence of severe mitral valve regurgitation and fast AF, which made TTE interpretation non-conclusive. Therefore, our patient underwent cardiac MRI (CMRI), confirming the aforementioned structural changes with the presence of HFpEF and diastolic dysfunction (Video 1).

**Video 1:** Cardiac magnetic resonance imaging (CMRI) of the patient The video shows bilateral atrial dilatation and left ventricular ejection fraction (LVEF) of 60% with diastolic dysfunction calculated by the biplane method. Severe free-flowing tricuspid and mitral valve regurgitation were also observed, with no evidence of pericardial effusion or ventricular abnormalities

A multidisciplinary team comprising an endocrinologist, a cardiologist, and an internist started her on carbimazole 40 mg and furosemide tablets 40 mg once daily, along with symptomatic treatment with propranolol 20-60 mg three times daily to be titrated according to the response.

A few days later, she was discharged in good condition with more controlled palpitations and tremors, and a follow-up visit was arranged with the endocrinology and cardiology clinics.

## Discussion

Thyroid hormones, particularly T3, affect cardiomyocytes through multiple mechanisms, including genomic regulation of cardiac gene expression, as well as non-genomic modulation of the plasma membrane or nuclear receptors in the cardiomyocytes [6]. Excessive thyroid hormones will lead to a hyperdynamic state by increasing the cardiac output and decreasing systemic vascular resistance. This can result in sinus tachycardia and AF due to the shortening of the refractory period of cardiomyocytes. Therefore, neglecting or underestimating these effects can take a heavy toll on the cardiovascular system over time, resulting in devastating cardiogenic shock and death [4,5,6].

Although TCM is a rare disease with an incidence of only 1% in patients with thyrotoxicosis, it is still a fatal type of dilated cardiomyopathy, and its prompt recognition is essential because it is a reversible cause of HF [6]. Recognizing TCM can be challenging due to the unexplained signs and symptoms of HF, making it a diagnosis of exclusion [5,6]. In cases of poorly controlled thyroid function, dilated cardiomyopathy typically starts with left ventricular hypertrophy and high cardiac output. However, as the disease progresses, biventricular dilatation and congestive HF with reduced ejection fraction become more common, as documented in the literature [5,7-12].

In the present study, instead of the expected features mentioned above, TTE revealed HFpEF and bilateral atrial dilatation with myxomatous changes of the atrioventricular valves, leading to significant secondary regurgitation both as a result of high intra-cardiac pressure and volume overload. This can be explained by elevated thyroid hormone levels that affect the hemodynamic components of the cardiovascular system in various ways [6,13]. Assessing diastolic dysfunction and left ventricular filling pressure in patients with HFpEF is essential. Although Doppler echocardiography is the best test for this purpose, it can sometimes be challenging to perform. Complicating factors such as AF and severe mitral regurgitation can make obtaining accurate results with Doppler echocardiography difficult. As a result, CMRI was performed on our patient [14]. CMRI is the gold standard non-invasive technique to differentiate between normal and abnormal relaxation patterns of the myocardium. It provides an opportunity to detect diastolic dysfunction early [15].

Since TCM is a diagnosis of exclusion, other differentials were excluded in our patient, including anemia, physical trauma, and Budd-Chiari syndrome as liver disease. The absence of thrombus or compression of the inferior vena cava ruled out Budd-Chiari syndrome, making passive hepatic congestion more likely, supported by tricuspid valve regurgitation and highly dilated right atrium [16]. It is worth noting that elevated ALP levels can be seen in up to 64% of patients with thyrotoxicosis per se or as a part of hidden autoimmune conditions that occur concurrently with Graves’ disease [17,18]. Tachycardia-induced cardiomyopathy (TIC) is still one of the primary differential diagnoses in such patients with undiagnosed AF for a long time, leading to cardiomyopathy [19]. TIC and TCM are intermingled in terms of pathophysiology and, partially, management since one of the latter's characteristic manifestations is sinus tachycardia and AF [4,5,6]. In fact, both diseases are diagnoses of exclusion and carry the same arrhythmogenic probability and prognosis; therefore, continuous ECG monitoring is useless for differentiation [19], creating a "two sides of the same coin" situation.

However, the presence of undiagnosed clinically overt thyrotoxicosis gave TCM the upper hand in the current study. In addition, the management plan focused on restoring the euthyroid state as quickly as possible since the related cardiovascular complications are often reversible, including HF and AF, especially in the early stages [20], which is unexpected if the patient was treated as a case of TIC with only antiarrhythmic drugs [6,19].

## Conclusions

It is crucial to maintain a high index of suspicion to detect and treat TCM promptly, and the management may require a multidisciplinary approach. This is not only important for prevention but also for reversing potentially life-threatening cardiovascular complications. Effective management of TCM demands collaborative care involving endocrinologists and cardiologists. Early recognition of atypical cardiac manifestations and tailored interventions based on patient response are imperative for a good prognosis.
